# How do creative assets affect overseas market entry modes of enterprises– the moderating role of internal and external factors

**DOI:** 10.1371/journal.pone.0296791

**Published:** 2024-02-07

**Authors:** Can Wang, Kangsheng Tao, Xiyuan Cui, Sushu Qin

**Affiliations:** 1 School of Business Administration, Hubei University of Economics, Wuhan, China; 2 Research center of Hubei logistics development, Hubei University of Economics, Wuhan, China; 3 School of Economics and Management, Enshi Vocational and Technical College, Enshi, China; 4 School of Business Administration, Zhongnan University of Economics and Law, Wuhan, China; 5 School of Economics and Management, Hubei Minzu University, Enshi, China; KAIST: Korea Advanced Institute of Science and Technology, KOREA, REPUBLIC OF

## Abstract

In the process of transnational investment management, the choice of entry mode is one of the key decisions, and creative assets will affect the choice of overseas market entry mode. However, few studies have analyzed how creative assets affect firms’ overseas market entry patterns. This paper takes 480 overseas investment data of 134 Chinese listed enterprises from 2012 to 2019 as research samples and uses the Logistic model to study the influence of creative assets owned by enterprises on their choice of the joint venture and wholly owned modes. At the same time, we examine the formal and informal institutional distance between home and host countries, and the moderating effect of firms’ own experience in the process of model selection. In addition, using the fuzzy-set qualitative comparative analysis (fsQCA) from the perspective of configuration to explore the different paths of overseas market entry mode. The research results indicate that marketing intensity and technical intensity of enterprises have a positive and significant impact on overseas investment patterns, that is, higher marketing intensity or technical intensity will prompt enterprises to preferentially choose wholly-owned mode. Formal institutional distance and experience can moderate the relationship between creativity and investment mode to some extent, while informal institutional distance has no significant moderating effect on creative assets and investment mode. Three configurations can induce firms to choose the wholly-owned mode, and only one configuration can induce firms to choose the joint venture mode. This study lays a theoretical foundation for enterprises to enter the overseas market.

## Introduction

The outbreak of COVID-19 has affected international investment cooperation and added many risks and challenges to Chinese enterprises “going global”. However, through the implementation of foreign policies such as the Belt and Road Initiative and the Regional Comprehensive Economic Partnership Agreement, the Chinese government has strengthened cooperation and exchanges with other countries and accelerated the integration of Chinese enterprises into the world. Thus, Chinese enterprises’ outward direct investment has bucked the trend. Among them, one of the main issues that enterprises need to consider in foreign investment is the choice of investment mode.

The outbound investment mode of an enterprise is an institutional arrangement [1]. The choice of entry mode is a cruical decision in the internationalization strategy of an enterprise, which affects the future management and operation of an enterprise in the host country. Existing studies have explored the factors influencing the choice of entry mode, such as the market factors in the host country [2, 3] and institutional factors in host countries [4, 5], home country institutional factors [6, 7], home market factors [8] relevant factors of enterprises themselves [9–11] and so on. [12, 13] prove that the nature of an enterprise’s proprietary assets is one of the important factors affecting the overseas market entry mode. However, as a kind of proprietary asset, the impact of creative assets on the overseas market entry mode is unknown.

The concept of “creative asset” was first proposed by Dunning in 1992. Creative assets are knowledge-based assets created through acquired effort based on natural resources and are a source of competitive advantage for organizations. Creative assets can be tangible, such as financial assets and distribution networks, or intangible, such as brands, goodwill, government relationships, and management skills. [14, 15]. The accumulation of creative assets is very difficult and requires long-term cultivation and huge investment. Due to transaction costs, knowledge transfer, and complementary resource demand, it will affect enterprises’ choice of market entry mode in the host country [16–19]. In addition to the assets owned by enterprises, in the process of internationalization, enterprises will inevitably encounter the differences between the home country and the host country in terms of external formal and informal systems such as laws, political systems, and customs, and the degree of such differences is “distance” [20]. Formal institutional distance and informal institutional distance will affect the choice of entry mode from the aspects of transaction cost, legitimacy, knowledge transfer efficiency, and so on. Enterprises must make matching decisions according to their actual situation and external institutional distance, to ensure their survival and lasting operation.

In addition, the experience of the host country will also affect the overseas market entry mode. The accumulation of experience enhances the enterprise’s understanding of the host country’s environmental culture, business practices, system norms, and other aspects, reduces uncertainty, and can form the accumulation advantage of the enterprise [21]. With experience, the management ability of overseas subsidiaries, the ability to predict business risks, and the ability to select partners will be improved, which may reduce transaction costs, and may choose external partners [22].

Therefore, based on 480 overseas investment activities of 134 enterprises from 2012 to 2019, this study analyzed the relationship between creative assets and enterprises’ overseas market entry mode and took into account the moderating effects of external factors, formal institutional distance, informal distance, and internal factors, firm experience. Further from the perspective of configuration, the paper explores the path of overseas market entry mode. The main contributions of this study are as follows. Firstly, the most significant contribution examines the relationship between creative assets and enterprises’ overseas market entry mode in the Chinese context, which enriches the relevant research on overseas market entry mode. Secondly, by examining the moderating effect of internal and external factors on the relationship between creative assets and the overseas market entry mode of enterprises, the research conclusion is helpful for enterprises to choose investment modes in different environments. Thirdly, the research results are of great significance in guiding management practice. In the light of empirical analysis, the research considers the potential interdependence of variables and asymmetric data relations and analyzes the preconditions for enterprises to choose different entry modes from the perspective of configuration, which makes up for the shortcomings of econometric models, providing a more detailed understanding of how enterprises choose overseas market entry modes and provide a theoretical basis for enterprises’ practice.

## Theory and hypotheses

**Fig 1:**
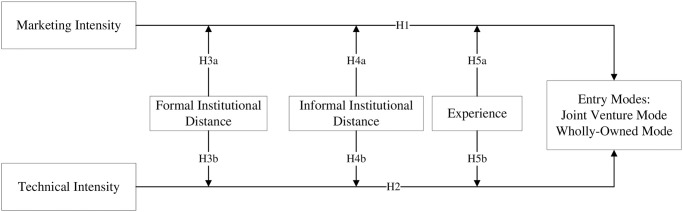
Proposed model.

This section provides a research model as in Fig 1. We also proposed related hypotheses and further explore the influential factors through empirical research.

### Creative assets and the enterprise entry mode

Creative assets can be divided into location-specific creative assets and firm-specific creative assets [23]. Locational creative assets are the unique creative assets of a certain location, such as technology, system, infrastructure, and other non-mobile assets, which can be shared by all enterprises, so they are also called locational advantages. The unique creative assets of an enterprise are the knowledge-based assets of an enterprise, such as brand, proprietary technology, management, and marketing channels, which are cultivated based on natural resources through long-term efforts, and have the characteristics of being unique to the company, difficult to imitate, untradable, and high transfer cost [14, 15]. In the existing literature, most studies on the specific asset variables at the enterprise level focus on marketing intensity and research and development intensity. Marketing intensity measures an enterprise’s investment in brand building, marketing channel network, proprietary assets, and accumulation of knowledge and experience. Technical intensity reflects an enterprise’s investment in proprietary technology, proprietary assets, and accumulation of knowledge and technical experience [24–26]. Therefore, based on other studies and the actual situation, this paper also chooses two dimensions of marketing and research and development variables to characterize creative assets respectively.

Marketing intensity refers to the resources and energy invested by enterprises in marketing strategy, as well as the intensity and strength of marketing activities [27]. Marketing is an important functional strategy of the enterprise, which introduces the enterprise to the market, lets the market accept the enterprise, establishes the image and reputation of the enterprise, and attracts customers for the enterprise, to make the enterprise gain profits [28]. The greater the marketing intensity of the enterprise, means that the enterprise attaches more importance to the overseas market and invests more resources and energy. First, the enterprise that has a high marketing intensity, can better realize the marketing strategy and promotion activities. While avoiding the friction and conflicts that may occur when cooperating with other enterprises, it needs a high degree of independent decision-making and independent operation rights. Secondly, enterprises with high marketing intensity need to occupy foreign markets as soon as possible, better control brand image and product quality, and avoid negative effects due to the reasons of partners. The wholly-owned mode helps enterprises better establish brand image and reputation. Third, enterprises with high marketing intensity invest more resources in technology, production, sales, marketing, and other aspects, and need to deploy resources more flexibly to better meet the overseas market demand and improve market competitiveness [29]. The wholly-owned mode is conducive to better integration of resources for enterprises. Not only that, the wholly-owned mode helps enterprises improve their innovation ability. Under the sole proprietorship model, enterprises can carry out research and development and innovation more independently, to better meet market demand and improve product competitiveness. Companies can manage and control risk more directly and avoid the risks and uncertainties that may arise when working with other companies. To sum up, the stronger the marketing intensity of the enterprise, the more inclined to choose the sole proprietorship mode to enter the overseas market. The sole proprietorship model can enable enterprises to fully control their management rights in overseas markets, achieve their strategic objectives, and help enterprises build brand image and reputation, integrate resources, improve innovation ability, and manage risks [30].

Technical intensity refers to the proportion of investment in research and development, reflecting the degree of investment in technological innovation [31, 32]. The improvement of technological strength of enterprises can promote the innovation and development of enterprises, improve the quality and efficiency of products and services, reduce costs, and enhance market competitiveness [33]. The greater the technical strength of the enterprise, means that the enterprise has invested more resources and energy in technology research and development and innovation [34]. In this case, enterprises are more inclined to choose the sole proprietorship model to enter the overseas market for the following reasons. First, when the technical intensity of the enterprise is large, its demand for technology protection also increases correspondingly. The sole proprietorship model can better protect the core technology and intellectual property of the enterprise, and avoid technology leakage or imitation. Under the sole proprietorship model, companies can better control the development, production, and sale of technology and products, thus ensuring the confidentiality and uniqueness of the technology. Second, the technical strength and innovation ability of enterprises have an important impact on the shaping of brand image. The sole proprietorship model can enable enterprises to better control brand image and product quality, to establish a good reputation and reputation in overseas markets. Through sole proprietorship, enterprises can focus more on brand building and marketing, and improve brand awareness and competitiveness. Third, the technological strength and innovation ability of an enterprise have an important impact on its future development. The sole proprietorship mode can enable enterprises to better inherit and carry forward their own technological advantages and innovative spirit, to achieve continuous technological updates and upgrades. Meanwhile, enterprises can focus on technology research and development and innovation, and continue to introduce more leading and competitive products and services. All in all, the greater the technical strength of the enterprise, it means that the enterprise has stronger strength and demand in the aspects of technological innovation and core technology protection. In this case, enterprises are more inclined to choose the sole proprietorship model to enter the overseas market. The wholly owned mode can better protect the enterprise’s core technology and intellectual property, build brand image, integrate resources, and inherent technical advantages, and manage risks [35].

In addition, enterprises entering the host country market may also have a demand for complementary local resources. If the enterprise has a strong motivation to develop new resources and the benefits brought by the host country’s partners are greater, the enterprise is willing to use a joint venture mode. On the contrary, if a company has a strong competitive advantage, such as high marketing intensity and strong technology, it does not need to seek assistance from local partners and can independently face risks and uncertainties, overcome the disadvantages of outsiders, and may choose the sole proprietorship mode [36]. Therefore, this paper proposes the following hypotheses:

**H1: The stronger the marketing intensity of enterprises, the more inclined they are to choose the wholly-owned mode to enter the host country market**.
**H2: The stronger the technical intensity of enterprises, the more inclined they are to choose the wholly-owned mode to enter the host country market**.

### The moderating effect of formal institutional distance

Formal institutional distance refers to the differences between countries in terms of mandatory and written regulations, mainly including the differences in various laws, regulations, and policies. Generally speaking, the formal system is clearly defined, and clearly coded, with the characteristics of formalization and formalization, easy to understand and implement, and enforced by the government department. Therefore, even without the help of local partners, enterprises can adjust themselves to meet the requirements of local formal regulations, go through the official approval process, and then obtain legitimacy. At this time, enterprises pay more attention to the parent company’s knowledge transfer and effective use of resources [37].

Due to the differences in language, culture, regulations, policies, and other aspects, the enterprise needs to invest more resources in market research and adaptation, when an enterprise enters a host country with a large institutional environment different from its own country. At this time, high marketing intensity can help enterprises better understand and adapt to the market demand and consumer behavior of the host country, and then choose the wholly owned mode. When enterprises face large institutional differences, they will assess risks more carefully and tend to choose deeper entry modes to reduce uncertainty and risk. High marketing intensity can enhance a company’s market perception and risk assessment capabilities, thereby helping companies make more informed decisions [38].

The knowledge and technology of high technical intensity enterprises are embedded in the organization, and the transmission process itself needs to go through the process of encoding, decoding, and re-encoding. Formal institutional distance may regulate the relationship between technical intensity and entry patterns by affecting the intensity of technology licensing and intellectual property protection [39, 40]. When the distance between formal institutions is relatively high, the knowledge structure of the parent company is inconsistent with the control structure of the host country, and the employees of the host country may resist the knowledge structure of the parent company, including technology, organizational process, management practice, etc. Therefore, the parent company and the host country partner need to carry out enterprise integration to make the technology of the integrated company and the parent company adapt to each other. It is also consistent with the local policy environment. Therefore, if an enterprise chooses the sole proprietorship model, it will have a higher degree of control over its overseas subsidiaries, reduce the cost brought by integration, suffer less external interference, and improve the probability of success of knowledge transfer and the survival rate of overseas subsidiaries [41].

Although the joint venture mode can bring benefits such as acquiring local knowledge and experience and reducing the risks caused by unfamiliarity, it is difficult and costly to select suitable partners and sign complex cooperation agreements in an unfamiliar host country environment. In addition, in the process of cooperation, partners are likely to produce speculative behaviors out of the need to obtain resources, which requires more control and coordination, and then the corresponding costs, plus the new costs of the parent company and the partner’s integration mentioned above. With the increase of formal institutional distance, the costs brought by these joint venture modes will exceed the benefits brought by the joint venture mode, and the difficulties encountered by enterprises in the operation process will exceed the demand for local resources, so the joint venture mode is no longer so advantageous. When choosing the sole proprietorship mode, enterprises can reduce the transaction costs caused by searching for partners, signing cooperation agreements and internal integration and coordination, effectively prevent the speculative behavior of partners, and make decisions faster, which is conducive to the survival and operation of enterprises in the host country market [42]. Therefore, this paper proposes the following hypothesis:

**H3a: Formal institutional distance positively moderates the relationship between marketing intensity and entry mode**.
**H3b: Formal institutional distance positively moderates the relationship between technical intensity and entry mode**.

### The moderating effect of informal institutional distance

Informal institutions mainly include the values, social norms, beliefs, and cultural cognition of a country or region, which are deeply embedded in the local social environment. The pressure of informal institutions is realized through imitation and normative mechanisms, without centralized coercive mechanisms, and enterprises need to weigh whether they need to follow the pressure brought by informal institutions [43]. At the same time, compared with the formal institute, the content of the informal system is more recessive and difficult to understand and grasp. Therefore, in the process of entering a new overseas market, the difficulty of obtaining the legitimacy of an overseas subsidiary will be higher than the situation with a larger distance from the formal system, because it is difficult for the subsidiary to obtain legitimacy by improving its conditions. At this time, obtaining legitimacy is the first goal that enterprises will take into account [44].

In addition, compared with the home market, the informal institutional distance increases the difficulties encountered by firms in acquiring knowledge of market opportunities in the host country and processing knowledge and increases the cost of information acquisition [45, 46]. As local employees follow different social norms, value systems, and beliefs, they may find it difficult to understand or accept the culture of the home country of the enterprise, and their expectations are more diversified, which may cause internal conflicts and increase the management costs of the organization. The effective transfer of the parent company’s internal management, procedures, and management practices, as well as the successful establishment, management, and maintenance of the relationship between the enterprise and the local management and stakeholders, will become difficult due to the increasing distance from the informal system [47, 48].

When businesses operate in different countries and regions, they face cultural differences. These differences come from values, social norms, belief systems, etc., which affect the communication and understanding between companies and local consumers and stakeholders [40, 49]. If a company’s marketing strategy is not compatible with the local culture, it will lead to a reduction in marketing intensity, and then reduce the speed of the company’s entry into the overseas market. For example, business practices in some countries or regions are more focused on interpersonal relationships and social networks, which can prompt companies to rely more on local networks for marketing activities and market access. In such cases, companies prefer to option for a joint venture model to better leverage local networks. Moreover, informal institutional distance reflects the differences in consumer psychology and brand image in different countries and regions, which affect the marketing strategies of enterprises in overseas markets. For instance, consumers in some countries or regions pay more attention to the localization and cultural adaptation of brands, which will prompt enterprises to pay more attention to the localization image and cultural adaptation of brands to adapt to local consumer needs and market conditions. In this case, enterprises may be more inclined to choose the joint venture model to better manage and shape the brand image.

When enterprises enter a new market, they will face technical risks and uncertainties, especially for highly technology-intensive enterprises. If the distance between local informal institutions is large, it will be more difficult for enterprises to understand and adapt to the local technological environment and market conditions [40], thus increasing the uncertainty between the intensity of technology and the mode of entry. In this case, enterprises prefer to choose the joint venture model to better control risks and uncertainties. If local informal institutions are more distant, companies need more time and resources to build and maintain trust and technology partnerships with local partners, suppliers, and customers [50]. In this case, companies will also choose the joint venture model to avoid potential conflicts with local partners and the uncertainty of technical cooperation. Because of the distance between informal institutions, the spread and application of technology may be limited, making it difficult for enterprises to benefit from local technology spillovers. In this case, enterprises choose the joint venture model to better control the spread and learning effects of technology and to better adapt to and take advantage of local technology and market conditions. Therefore, this paper proposes the following hypothesis:

**H4a: Informal institutional distance negatively moderates the relationship between marketing intensity and entry mode**.
**H4b: Informal institutional distance negatively moderates the relationship between technical intensity and entry mode**.

### The moderating effect of experience

When enterprises lack sufficient experience, it is difficult to accurately evaluate the market and competitive environment, as well as develop effective marketing strategies. In this case, enterprises are more inclined to choose the sole proprietorship model to reduce decision-making risk and better control marketing activities. Companies with overseas investment experience can perceive less uncertainty, have stronger forecasting ability, and know how to deal with unexpected problems in the future [51], thus reducing the need for the sole proprietorship model. When entering a new market, enterprises need a certain learning and adaptation process to better understand the local market and competitive environment. Businesses with experience can learn and adapt quickly, and effectively use resources to develop marketing strategies. In such cases, companies may prefer a joint venture mode to better control marketing resources and avoid potential conflicts with local partners [52].

When entering a new market or adopting new technology, the lack of experience makes enterprises lack sufficient understanding of the local technology environment, market demand, and competition situation, which increases the uncertainty between the technology intensity and the entry mode [53]. To reduce this uncertainty, enterprises choose the sole proprietorship model to better control risk and adapt to the technological environment. Enterprises with overseas investment experience, familiarity with the foreign technical environment, and the need, to learn faster, research and development of new technologies are more willing to enter the overseas market through joint ventures [54]. In some cases, companies may need to establish technical partnerships with local research institutions, suppliers, or customers to access resources such as technical support and market access. However, the lack of experience may make it difficult to establish and maintain effective cooperative relationships with local partners, which in turn affects the technical intensity of enterprises and the choice of entry mode. Experienced enterprises can effectively absorb and apply new technologies, and then affect the technology intensity and the choice of entry mode of enterprises. To better adapt to and take advantage of local technology and market conditions, enterprises may be more inclined to choose the joint venture model.

Furthermore, establishing partnerships is inherently difficult and requires mutual support and trust among partners [55]. Successful joint ventures are usually established based on existing relationships rather than initial collaborations, maintaining long-term relationships through contracts that prevent the other party from engaging in competitive activities. However, companies come from different countries and have different resource capabilities, and their strategic goals and organizational culture may not necessarily match each other. This divergence may harm the profitability and stability of joint ventures. Experienced enterprises, as they have adapted to the environment and have more knowledge, can better identify and select suitable partners, sign cooperation agreements more easily, authorize partners to control some market operations, and overcome coordination problems, thus making the joint venture mode more visible and stable [56]. Therefore, this paper proposes the following hypothesis:

**H5a: Experience negatively moderates the relationship between marketing intensity and entry mode**.
**H5b: Experience negatively moderates the relationship between technical intensity and entry mode**.

## Methods

### Data collection

According to the Classification of National Economic Industries, Chinese industries can be divided into three categories, the primary industry is agriculture. The second industry is the processing and manufacturing industry. The tertiary industry is the service industry, which specifically refers to other industries except the primary industry and the secondary industry. Agriculture and manufacturing have been studied by many scholars, but few scholars take the service industry as the research object. Therefore, this paper chooses the tertiary industry as the research object. The tertiary industry is the service industry, i.e. the industry that does not produce material goods, which mainly includes the transportation, storage, and postal industry, information transmission, computer services and software industry, wholesale and retail trade, public administration and social organizations, and international organizations. Based on this classification standard, this paper compares the list of listed companies and corporate annual reports to screen the service enterprises that have made overseas investments in the Shanghai and Shenzhen stock markets from 2012 to 2019 and preliminarily obtains the list of target enterprises. Meanwhile, the basic information of the enterprise and the year and content of overseas investment activities are collected. In the actual operation process, some enterprises choose regions such as Hong Kong, Macao, Virgin Islands, Cayman Islands, and Bermuda to obtain preferential policies such as tax exemption, rather than to achieve real international development. Therefore, this paper excludes investment enterprises in such regions.

Through the collection of data, 480 overseas investment activities of 134 enterprises from 2012 to 2019 were finally obtained. The distribution of these 480 items is shown in Table 1. From the geographical distribution of investment projects, investment projects are mainly distributed in 51 regions, of which North America, Europe, and Asian countries reach 416, accounting for more than 80. Asian countries had the largest number of projects, reaching 166, accounting for 34.58%Ṫhe country with the largest number of projects is the United States, reaching 134 projects, accounting for 27.92% of the total sample. Additionally, this paper collects other company-level data from corporate annual reports, prospectuses, the CSMAR database, and the official website of the World Bank.

**Table 1:** Geographical distribution of investment projects.

Region	Number	Percent (%)	Region	Number	Percent (%)
Canada	4	0.83	Luxembourg	11	2.30
USA	134	27.92	Romania	1	0.21
Mexico	2	0.42	Peru	2	0.42
Australia	44	9.17	Portugal	1	0.21
New Zealand	5	1.04	Sweden	3	0.63
Congo	2	0.42	Switzerland	4	0.83
Kenya	2	0.42	Sri Lanka	2	0.42
South Africa	4	0.83	Turkey	2	0.42
Nigeria	1	0.21	Spain	6	1.25
Uganda	1	0.21	Italy	1	0.21
Brazil	2	0.42	United Kingdom	23	4.79
Colombia	2	0.42	United Arab Emirates	6	1.25
Chile	1	0.21	Philippines	1	0.21
Albania	1	0.21	Korea	18	3.75
Ireland	2	0.42	Cambodia	1	0.21
Austria	1	0.21	Malaysia	19	3.96
Belgium	3	0.63	Myanmar	4	0.83
Poland	1	0.21	Japan	19	3.96
Denmark	1	0.21	Thailand	4	0.83
Germany	18	3.75	Brunei	1	0.21
Russia	6	1.25	Singapore	60	12.50
France	6	1.25	India	7	1.46
Finland	3	0.63	Indonesia	8	1.67
Netherlands	11	2.29	Vietnam	2	0.42
Czech	1	0.21	Israel	3	0.63
Taiwan(China)	13	2.71			

### Variable measurement

#### Dependent variables

This paper takes the mode of entering the foreign market as an explanatory variable, which can be divided into two modes: wholly owned and joint venture. The relevant data to measure the wholly owned and joint venture mode comes from the company’s annual report, prospectus, and other public materials. According to the study of [57], when the share ratio is between 10% and 90%, it is a joint venture and is denoted as 0. When the equity ratio exceeds 90%, it is wholly owned and is denoted as 1. Furthermore, we remove samples where the share ratio is less than 10%.

#### Independent variables

*Marketing intensity (MI)*. Marketing intensity measures an enterprise’s unique advantages, knowledgeability, and proprietary assets in marketing. Some scholars use the ratio of advertising investment to operating income to measure this index, but advertising investment is not an item that must be published in the annual reports of listed companies in China. This paper refers to the practice of [58]. Marketing intensity is expressed using the ratio of sales expenses to revenue. Given that investment decisions are made before the investment activity itself, data for the previous year in which the investment activity occurred are used in the calculation.

*Technical intensity (TI)*. Technical intensity is the most widely used measure of a firm’s unique technological strengths, knowledge capabilities, and proprietary assets [59, 60], which is equal to the ratio of annual technical investment to current operating income. Given that investment decisions are made before the investment activity itself, data for the previous year in which the investment activity occurred are used in the calculation.

#### Moderator variables

*Formal institutional distance (FD)*. The World Bank’s Global Governance Index provides overall and individual governance indicators for more than 200 countries and regions from 1996 to 2017, covering six dimensions: voice and accountability, political stability and absence of violence, government efficiency, regulatory quality, rule of law, and control of corruption. The indicators, which combine the views of a large number of business, citizen, and expert respondents, are compiled from more than 30 separate data sources from various survey agencies, think tanks, non-governmental organizations, international organizations, and private companies. This paper chooses the Global Governance Index to measure formal institutional distance. Considering that the investment decision is made before the investment activity itself, this paper uses the data of the year before the overseas investment activity of the current year, that is, the investment activity occurred in the t year, and the data of the t-1 year is used to calculate the formal institutional distance. This paper combines [20, 61] to calculate the formal institutional distance between the host country and the home country.

$$FD_{j}=\frac{\sum_{i=1}^{6}\{{\left(\omega_{i}-\omega_{ic}\right)}^{2}/\xi_{i}\}}{6}$$

Where *ω*_*i*_ denotes the institutional score of country *j* under the *i* dimension, *ξ*_*i*_ denotes the score variance under this dimension, *ω*_*i*_*c* denotes the institutional score of China under this dimension, *FD*_*j*_ denotes the formal institutional distance between country *j* and China.

*Informal institutional distance (IFD)*. Based on the research of [62, 63], this paper uses cultural distance to measure informal institutional distance. Hofstede divides national culture into six dimensions, including power distance, individualism and collectivism, masculinity and feminism, uncertainty avoidance, long-term orientation, and abuse and restriction. Considering that the investment decision is made before the investment activity itself, this paper uses the data of the year before the overseas investment activity of the current year, that is, the investment activity occurred in the *t* year, and the data of the *t* − 1 year is used to calculate the informal institutional distance. This paper refers to previous research results [20, 63], the detailed calculation formula is as follows:

$$IFD_{j}=\frac{\sum_{i=1}^{6}\{{\left(\chi_{i}-\chi_{ic}\right)}^{2}/\delta_{i}\}}{6}$$

Where *χ*_*i*_ denotes the cultural system score of country *j* under the *i* dimension, *δ*_*i*_ denotes the score variance under this dimension, *χ*_*ic*_ denotes the cultural score of China under this dimension, and *IFD*_*j*_ denotes the informal institutional distance between country *j* and China.

*Experience*. Based on the practice of [64], experience in the host country market is set as a dummy variable. When the enterprise has not previously invested in the host country market, it is recorded as 0. When the enterprise has previously invested and operated in the host country market, it is recorded as 1.

#### Control variables

*Direct foreign investment (FDI)*. The market attraction of the host country has a certain influence on the choice of the enterprise’s entry mode. The more attractive the market, the more foreign investment in the host country, which means that a country can offer lower asset transfer costs, thus attracting investors. There is a positive relationship between market attractiveness and equity patterns [65]. Therefore, this paper selects the FDI of the host country in the year before the year in which the investment activities take place to measure the market attraction of the host country and uses its logarithm value in the calculation.

*Size*. Enterprise size is crucial for the resource allocation of enterprises [8]. The large scale may mean that enterprises have more resources, which also means organizational inertia. Therefore, this paper chooses size as a control variable. The operating income of the year before the year in which the investment activities occurred is used to measure the size of the company, and its logarithm value is used in the calculation.

*Age*. The age of the enterprise refers to the difference between the year before the investment activity of the enterprise and the year of establishment. Enterprise age is one of the factors influencing the choice of enterprise entry mode [66]. Enterprise age means enterprise management experience and resource scale but also means structural inertia. This paper also takes the age of the firm as the control variable. The data comes from the CSMR database.

### Regression model

Considering that the explained variables are binary, the LOGISTIC binary regression model was used for empirical analysis. In the variable setting, when the enterprise chooses the wholly owned mode, the value is 1, when the enterprise chooses the joint venture mode, the value is 0. Therefore, in the regression process, the positive regression coefficient indicates that with the increase of explanatory variables, enterprises are more likely to enter an overseas market through the wholly owned mode. The negative regression coefficient indicates that with the increase of explanatory variables, an enterprise is more likely to enter an overseas market through the joint venture mode. To get a better effect of empirical results, we first centralize all variables other than dummy variables before regression analysis and then use the centralized data for regression analysis. The specific regression model is as follows:

$${EM}_{i}^{t}=\alpha_{0}+\beta_{1}\times MI_{i}^{t-1}+\beta_{2}\times TI_{i}^{t-1}+\beta_{3}Control+\varepsilon_{i}$$

$${EM}_{i}^{t}=\alpha_{0}+\beta_{1}\times MI_{i}^{t-1}+\beta_{2}\times TI_{i}^{t-1}+\beta_{3}Control+\beta_{4}MV_{i}^{t-1}\times DV_{i}^{t-1}+\varepsilon_{i}$$

Where *α*_0_ denotes constant, *ε*_*i*_ denotes random error, *i* represents the enterprise, and *t* represents the year. *EM* represents the enterprise’s overseas investment entry modes. *MI* and *TI* represent marketing intensity and technical intensity respectively. Control represents the control variables. *MV* represents the moderator variables. *DV* represents the independent variables.

## Data analysis and results

### Descriptive statistics and correlation analysis

The final data sample used for empirical testing in this study includes 134 enterprises conducting a total of 480 overseas investment projects between 2012 and 2019. The specific distribution is shown in Table 1. The projects are distributed across 51 regions, with the United States, Singapore, and Australia being the top three, with 134, 60, and 44 investment projects respectively. Out of these 480 projects, 359 project activities belong to the wholly-owned mode, while 121 project activities belong to the joint venture mode. Table 2 describes the main independent variables, moderating variables, and control variables. From Table 2, it is evident that the average value of entry mode is 0.747. The average values for marketing intensity and technical intensity are 0.089 and 0.046 respectively. The average values for formal institutional distance and informal distance are 3.269 and 2.688 respectively, with standard deviations of 1.404 and 1.469. This indicates that there are significant differences in the regions where the companies are located.

**Table 2:** Sample characteristics.

Variable	Mean	S.D.	Min	Max
Entry mode	0.747	0.434	0	1
Marketing intensity	0.089	0.117	0	0.577
Technical intensity	0.046	0.093	0	0.829
FD	3.269	1.404	0.130	5.984
IFD	2.688	1.469	0	7.640
Experience	0.200	0.399	0	1
FDI(log)	4.420	1.102	0	5.668
Size	8.217	3.240	0	11.482
Age	17.65	10.091	1	64

Table 3 presents the correlation coefficients among the variables. It is observed from Table 3 that there is a positive correlation between entry mode and both marketing intensity and technical intensity, providing preliminary support for hypotheses 1 and 2. The maximum value of the correlation coefficient between marketing intensity and technical intensity is 0.509 and it is significant at 0.01 level. In addition, this study uses SPSS software to calculate the variance inflation factor of the variables, and it is found that the variance inflation factor of the variables is less than 3, and it can be assumed that there is no multicollinearity between the variables.

**Table 3:** Variable correlation analysis.

Variable	1	2	3	4	5	6	7	8
Entry mode	1							
Marketing intensity	0.103*	1						
Technical intensity	0.133**	0.509**	1					
FD	-0.004	-0.037	-0.050	1				
IFD	0.073	-0.015	-0.057	0.290**	1			
Experience	0.120**	0.051	0.062	0.022	0.180**	1		
FDI(log)	0.020	0.016	-0.023	0.112*	0.238**	0.169**	1	
Size	-0.098*	0.220**	0.096*	0.096*	0.116*	-0.007	0.169**	1
Age	0.013	-0.088	-0.013	-0.005	-0.030	0.033	-0.001	0.331**

*p <0.05,

**p <0.01.

### Regression analysis

The empirical results of the regression model are summarized in Table 4. Model 1 includes only the control variables host country FDI, firm age, and firm size. Model 1 reveals that the p-value for the Omnibus test is 0.072, while the p-value for the Hosmer-Lemeshow test is 0.168. In the regression results of Model 1, the p-values of firm age and host country FDI are all greater than 0.1, indicating that there is no significant direct correlation between these two variables and entry mode. The p-value of firm size is 0.015, which is less than 0.05, and the coefficient is -0.1, which indicates that there is a negative correlation between firm size and entry mode, that is, the larger the size of the firm, the more the firm tends to choose the joint venture mode to enter the overseas market. Based on Model 1, the study added two explanatory variables (marketing intensity and technical intensity) as creative assets. Model 2 demonstrates statistical significance as indicated by the Omnibus test’s p-value being less than 0.05, while the Hosmer-Lemeshow test shows a p-value significantly greater than 0.05. Moreover, Cox & Snell R-squared is 0.048 and Nagelkerke R-squared is 0.072, which are significantly larger than the values in Model 1, and thus the overall fit of the model is also better than that of Model 1. In the regression results of model 2, the p-value of marketing intensity is 0.092 and the coefficient is 2.219, which indicates that there is a positive correlation between marketing intensity and entry mode at the 0.1 significance level. If the marketing intensity of a company is greater, firms are more likely to choose the wholly owned mode. The p-value of technical intensity is 0.023 and the coefficient is 4.850, indicating a significant positive correlation between technical intensity and entry mode at the 0.05 level. When the greater the technical intensity of an enterprise, the more likely it is to choose the wholly owned mode of entry into overseas markets. Therefore, both Hypothesis H1 and Hypothesis H2 are supported.

**Table 4:** Regression model results.

Variable	Model 1	Model 2	Model3	Model 4	Model 5	Model 6	Model 7	Model 8	Model 9
Marketing intensity(MI)		2.219*	2.132	2.483*	2.135	2.322*	2.151	3.526**	2.223*
Technical intensity(TI)		4.850**	5.067**	5.545**	5.058**	5.181**	5.519**	5.693**	6.959**
Age	0.014	0.019	0.019	0.019	0.036	0.020	0.019	0.020	0.018
Size	-0.100**	-0.126**	-0.129**	-0.128**	-0.129**	-0.129**	-0.128**	-0.136**	-0.131**
FDI	0.084	0.106	0.036	0.041	0.019	0.030	0.035	0.048	0.030
FD			-0.017	0.023	-0.013	-0.020	-0.018	-0.022	-0.015
IFD			0.137*	0.131*	0.137*	0.162*	0.165*	0.146*	0.146*
Experience			0.650**	0.651**	0.649**	0.632*	0.633*	0.613*	0.565*
FD * MI				1.607*					
FD * TI					0.214				
IFD * MI						0.735			
IFD * TI							1.129		
Experience * MI								-6.090**	
Experience * TI									-6.770*
constant	0.962***	0.975***	0.885***	0.908***	0.884***	0.884***	0.899***	0.921***	0.930***
Omnibus test	0.072	0.000	0.000	0.000	0.000	0.000	0.000	0.000	0.000
Cox & Snell *R*^2^	0.014	0.048	0.065	0.072	0.065	0.067	0.066	0.075	0.070
Nagelkerke *R*^2^	0.021	0.072	0.097	0.107	0.097	0.098	0.098	0.110	0.104
Hosmer-Lemeshow	0.168	0.797	0.553	0.285	0.340	0.265	0.467	0.094	0.285

*** p<0.01,

** p<0.05,

* p<0.1.

Based on Model 2, three moderating variables, formal institutional distance, informal institutional distance, and experience, are introduced into Model 3 to test the boundary condition about the relationships between creative assets and entry mode. In model 3, the p-value of the Omnibus test is less than 0.05 and the p-value of the Hosmer-Lemeshow test is greater than 0.05, thus still indicating a significant model. In terms of model fit, Cox & Snell R-squared is 0.065 and Nagelkerke R-squared is 0.097, which is significantly larger than Model 2, so the fit of Model 3 is also improved. The coefficient of marketing intensity is 2.132 and the p-value is 0.11. The coefficient of technical intensity is 5.067 and the p-value is 0.022, which is less than 0.05. Based on Model 3, the interaction terms of formal institutional distance with marketing intensity and formal institutional distance with technical intensity were introduced into Models 4 and 5, respectively. The results show that the p-value of the interaction term between formal institutional distance and marketing intensity is 0.054 (less than 0.1), with a coefficient of 1.607, which indicates that formal institutional distance positively moderates the relationship between marketing intensity and entry mode, that is, the positive relationship between marketing intensity and entry mode is enhanced in the case of larger formal institutional distance, and Hypothesis 3a is supported. However, the p-value of the interaction term between formal institutional distance and technical intensity is 0.875 (greater than 0.1) with a coefficient of 0.214, which suggests that the moderating effect of formal institutional distance on the relationship between technical intensity and entry mode is not significant, and hypothesis 3b is not supported.

Based on Model 3, the interaction term between informal institutional distance and marketing intensity, and the interaction term between informal institutional distance and technical intensity were introduced sequentially into Models 6 and 7. In both Model 6 and Model 7, the p-value of the Omnibus test is less than 0.05 and the p-value of the Hosmer-Lemeshow test is more than 0.05. Thus, both models were significant. The results show that the p-value of the interaction term between informal institutional distance and marketing intensity is 0.436 (greater than 0.1) and the coefficient is 0.73. The p-value for the interaction term of informal institutional distance and technical intensity is 0.485 (greater than 0.1) with a coefficient of 1.129. This indicates that the moderating effect of informal institutional distance on the relationship between marketing intensity and entry mode, and the relationship between technical intensity and entry mode is insignificant. Therefore, hypotheses 4a and 4b are not supported.

Based on Model 3, the interaction term between experiences and marketing intensity, and the interaction term between experiences and technical intensity were introduced sequentially into Models 8 and 9. In both Model 8 and Model 9, the p-value of the Omnibus test is less than 0.05 and the p-value of the Hosmer-Lemeshow test is more than 0.05. Thus both models were significant overall. The p-value of the interaction term between experience and marketing intensity in Model 8 is 0.022 (less than 0.05) with a coefficient of -6.090. This indicates that the interaction term is significantly negatively correlated with entry mode at the 0.05 level of significance and that experience negatively moderates the relationship between marketing intensity and entry mode. The positive relationship between marketing intensity and entry mode is weakened when firms are experienced. Therefore, hypothesis 5a is supported. In Model 9, the p-value of the interaction term between experience and technical intensity is 0.078 (less than 0.1), with a coefficient of -6.770. This demonstrates that experience negatively moderates the relationship between technical intensity and entry mode. The positive relationship between technical intensity and entry mode is weakened when firms have overseas experience. Therefore, hypothesis 5b is supported. In addition, this study further controls for year and industry fixed effects, and the results are consistent with the Table 4, indicating that the year and industry do not affect the hypothesized results, and the research results are robust.

### Fuzzy-set qualitative comparative analysis

Through empirical analysis, we find that marketing intensity and technical intensity can significantly affect the choice of enterprises’ overseas investment mode. In addition, cultural institutions and enterprises’ experience in overseas investment also affects the choice of enterprises’ overseas investment mode. However, the previous model only explored the independent marginal impact relationship between the independent and dependent variables, obtaining a uniformly symmetric relationship between the independent and dependent variables [67], and the combined effect between the antecedent variables was not analyzed. Therefore, the fuzzy-set qualitative comparative analysis(fsQCA) was used to explore the configuration effect between the antecedent variables and to analyze the path of corporate investment model selection. The fsQCA combines the respective strengths of qualitative and quantitative analyses and can address the limitations of structural equation modeling and multiple linear regression, explain the interdependent complex effects of independent variables, and answer causal complexity questions such as multiple concurrent causality and causal asymmetry [68].

Unlike traditional regression methods, the fsQCA is based on the principles of Boolean algebra, which allows in-depth analysis of the different combinations of conditional variables that produce outcomes, reveals the different causal pathways, and breaks through the limitations of traditional regression statistics in terms of exploring the ‘joint effect’ and ‘interaction effect’ of the influencing factors [69, 70]. Data must be calibrated before analysis can be performed. In this study, fsQCA 3.0 software was used for data calibration [71]. The entry mode and experience variables are dummy variables. Therefore, they do not need to be calibrated. Other variables refer to the study by [72] using the mean of the variables intersected. The specific calibration anchors are listed in Table 5.

**Table 5:** Calibration anchor points.

Variable	Anchor points		
	full membership	maximum uncertainty	non-membership
Marketing intensity	0.577	0.089	0
Technical intensity	0.829	0.046	0
Formal institutional distance	5.984	3.269	0.130
Informal institutional distance	7.640	2.688	0

After the calibration of the data, this study proceeds in turn with the necessity analysis and the configuration analysis. Table 6 shows the results of the necessity analysis. From Table 6, the consistency of all variables is less than 0.9, indicating that no conditions are necessary to cause the results [73]. It can be argued that neither the wholly-owned mode nor the joint venture mode is determined by a single fixed variable.

**Table 6:** Calibration anchor points.

Condition	Wholly owned mode		Joint venture mode	
	Consistency	Coverage	Consistency	Coverage
Experience	0.225	0.852	0.115	0.147
∼Experience	0.774	0.722	0.884	0.277
MI	0.324	0.785	0.261	0.214
∼MI	0.675	0.730	0.738	0.269
TI	0.251	0.794	0.193	0.205
∼TI	0.748	0.733	0.806	0.266
FD	0.531	0.746	0.535	0.253
∼FD	0.468	0.749	0.464	0.250
IFD	0.464	0.768	0.416	0.231
∼IFD	0.535	0.731	0.583	0.268

∼ denotes the low levels of a condition.

As a result of configuration analysis, complex, parsimonious, and intermediate solutions of varying simplicity are generated. The intermediate scheme combines the theoretical knowledge of the researcher with the analysis of the case, and the conclusions obtained are more revealing and generalizable and have been adopted by many scholars in studies using the fsQCA analysis method [74]. For this reason, the results of the intermediate solution are reported in the following analyses of this study. Meanwhile, this study refers to the study of [72] and takes the conditions that appear in both the parsimonious and intermediate solutions as the core conditions and defines all the conditions that appear in the intermediate solution but are excluded by the parsimonious solution as the peripheral conditions.

According to Ragin’s criteria, the consistency level was set to 0.8 and the frequency threshold was set to 2. The results of sufficient conditions are shown in Table 7. Overall, the level of overall solutions is higher than the level of 0.75 suggested by Ragin [69]. Therefore, the results are valid. Meanwhile, overall solution coverage reaches 0.181. It shows that these three paths explain to a large extent why firms choose to invest in an independent model. From the intermediate solution, companies have a preference for the wholly-owned investment mode in the following three scenarios. The first solution is a wholly owned mode consisting of high investment experience and low marketing intensity, with a group consistency of 0.846. The results show that when companies have previous experience investing overseas and have low brand influence and visibility, they also choose the wholly owned mode to integrate more quickly into the overseas market.

**Table 7:** Analysis of sufficient conditions.

Conditions	Wholly owned mode		
	Solution 1	Solution 2	Solution 3
Experience	●	●	●
Marketing intensity	⊗		
Technical intensity			•
Formal institutional distance			⊗
Informal institutional distance		•	
Consistency	0.846	0.881	0.831
Raw coverage	0.152	0.127	0.045
Unique coverage	0.049	0.016	0.002
Overall Solution Consistency		0.851	
Overall Solution Coverage		0.181	

The black circles represent high-level conditions and the cross circles represent low-level conditions. Larger circles represent core conditions, smaller circles represent peripheral conditions.

The second solution is high investment experience and high informal institutional distance creates a wholly owned mode with a maximum solution consistency of 0.881. The results show that when firms have experience in overseas investment and there are large cultural differences between countries, they still choose the wholly owned mode to minimize the hidden costs associated with cultural differences. A third solution is high foreign investment experience, high technical intensity, and low formal institutional distance, which also creates a wholly owned mode. If the company has overseas investment experience and strong technical intensity, and the company’s investment regulations and related laws are not very different from those in China, the company can quickly understand the investment laws and regulations, reduce the investment cost and reduce the risk of the company’s intellectual property rights being infringed, it will choose the wholly owned mode. In addition, this study is also an analysis of the configuration of the joint venture investment mode. The results show that when firms have no experience in overseas investment, marketing intensity, and technical intensity are low, and there is a large difference in distance from formal institutions and culture, they will prioritize the joint venture investment mode for overseas investment. All in all, overseas investment experience is the key for enterprises to choose the wholly-owned investment mode, while the laws and regulations and cultural differences of the investing countries are also important factors to be taken into consideration.

## Discussion and conclusion

This study takes 480 overseas investment data from 134 Chinese listed service industry enterprises in the Shanghai and Shenzhen stock markets from 2012 to 2019 as the research object and uses marketing intensity and technical intensity to characterize the creative assets of enterprises. The study investigates the impact of the creative assets owned by enterprises on their choice of joint venture/wholly owned modes. At the same time, it examines the moderating effect of the company’s internal factors-self-experience, external factors-formal institutional distance, and informal institutional distance. The results showed that the marketing intensity and technical intensity of enterprises have a positive and significant impact on overseas investment modes, that is, when the marketing intensity or technical intensity of enterprises is high, it will encourage enterprises to prioritize independent investment models. Formal institutional distance and experience can moderate the relationship between creativity and investment patterns to a certain extent, while informal institutional distance has no significant moderating effect on the relationship between creative assets and investment patterns. Surprisingly, this conclusion is inconsistent with the conclusion of [40]. [40] proved that informal institutional distance and formal institutional distance positively moderated the relationship between proprietary technology and entry mode selection. The possible reason is that the new technologies owned and developed by enterprises with high technological intensity are at the forefront of the world, and are not subject to institutional and cultural constraints. Huawei, for example, which adheres to independent research and development, is not susceptible to changes in the external environment and political environment to change the choice of foreign investment mode. In addition, based on empirical analysis, this research also uses the fuzzy-set qualitative comparative analysis method to explore the different paths of enterprises to choose the entry mode. This study breaks through the limitation of the offline relationship between variables and explores the combination effect between causal variables from the perspective of configuration, which makes up for the shortcomings of current research. The results show that three configurations can promote firms to choose independent investment mode, and only one configuration can promote firms to choose joint venture mode.

### Theoretical contributions

Firstly, this study enriches the research on creative assets and overseas investment by enterprises. The research results indicate that there is a positive correlation between creative assets and entry mode choices, that is, the greater the marketing intensity of a company, the more inclined it is to choose a wholly owned mode to enter the host country market; The greater the technical intensity of enterprises, the more inclined they are to choose the wholly owned mode to enter the host country market. To prevent the leakage or misuse of their creative assets, the efficiency of knowledge transfer, and the decrease in demand for complementary resource acquisition, enterprises will choose to use the sole proprietorship mode to enter the host country market. Secondly, considering both internal and external factors of the enterprise, the boundaries of the study on overseas market entry modes of enterprises have been further expanded. The results indicate that internal factors negatively regulate the relationship between creative assets and enterprise entry patterns, that is, with experience, the positive relationship between marketing intensity or technical intensity and entry patterns will be weakened. In the absence of experience, it is difficult for enterprises to evaluate the performance of their host country partners, and the risks and costs associated with the service industry’s resource commitment level are low, while the risks and costs of maintaining high control methods are low. Therefore, enterprises tend to choose the wholly owned mode. But for experienced companies, the risk prediction ability of managers has been improved, which can help companies deal with various problems in the future cooperation process and reduce various transaction risks and costs. In addition, experienced enterprises are also more able to choose suitable partners and have more management capabilities in cooperative relationships, which will also encourage them to choose a joint venture mode. The external factor, formal institutional distance, positively regulates the relationship between marketing intensity and enterprise entry mode, that is, when the formal institutional distance is large, the positive relationship between marketing intensity and entry mode will be strengthened. The possible reason is that China has always been advocating win-win cooperation and building a community of shared future for mankind, the company already has a certain accumulation of technology and patents and is still willing to use the method of sharing equity to make progress and create value with partners. Finally, this study utilizes fuzzy-set qualitative comparative analysis to explore the path of enterprise entry mode selection, opening up new research perspectives for enterprise overseas investment mode selection. The results show that three paths can encourage enterprises to choose wholly owned modes, they are high investment experience and low marketing intensity, high investment experience and high informal institutional distance, high overseas investment experience and high technical intensity, and low formal institutional distance. The research results have laid a theoretical foundation for enterprises to choose appropriate overseas market entry modes and pointed out new directions.

### Managerial implications

Firstly, enterprises should actively cultivate their core competitiveness and increase the accumulation of creative assets. Enterprises need to improve their awareness of innovation, focus on cultivating creative assets, formulate reasonable and practical development strategies, and improve their competitiveness by improving the quality of talents, increasing technical investment, and expanding marketing capabilities. At the same time, capable enterprises can also learn from the creative assets of developed countries through cross-border mergers and acquisitions, new investment, and actively participate in international competition, promoting their progress and development through competition. Secondly, the formal institutional is clearly defined, easy to understand and implement by enterprises, and enforced by government departments. For enterprises, it is easy to grasp the requirements of the formal institution. Therefore, when conducting overseas investment activities, enterprises should understand the stability and differences of the host country’s laws, policies, politics, and other systems in advance through research reports from international business consulting agencies, investment guidelines issued by government departments, and actively adjust themselves according to their requirements to adapt to their norms. The informal institutional includes the values, social beliefs, and cultural customs of a country or region, with characteristics of concealment, difficulty in understanding, and mastery, deeply embedded in the local social environment. Although the implementation of informal institutional is ensured by external mandatory mechanisms, enterprises also need to carefully consider and proactively understand and learn, such as by hiring local employees, training employees to learn the local language, or selecting Chinese managers with work and learning experience in the host country to reduce the impact of differences, increase understanding of the informal environment in the host country, and gradually integrate into the local market to gain legitimacy. Third, the experience of enterprises can improve the ability of Chinese enterprises to perceive and respond to external risks, and improve the management capabilities of overseas subsidiaries of enterprises, risk prediction ability and partner selection ability can ultimately improve the survival rate and business performance of enterprises in overseas markets. Therefore, enterprises should pay attention to the accumulation of international experience. For inexperienced Chinese companies, although they can choose the sole proprietorship mode because they want to improve internal management efficiency and reduce various costs arising from cooperation issues, they can still choose to appoint managers with rich overseas working experience or obtain knowledge from peers. Enterprises with experience in transnational operations and multinational companies operating in the same host country learn from the experience and acquire relevant experience in transnational operations to lay a foundation for their business development in the host country.

### Limitations and future research

Some limitations highlight the need for future research. First, apart from wholly-owned and joint ventures modes, there may also be a variety of investment modes, such as sole proprietorship—joint venture mode, high equity—low equity mode, etc. Future research can classify and discuss different modes. Second, this study discusses the impact of creative assets on entry mode, but the mechanism of this impact is still unclear. We can further consider adding intermediary variables such as technological innovation, human capital, and peer competition to further explore the mechanism of creative assets in entry mode. Third, the secondary data used in this study cannot fully reflect the choice of overseas entry mode of an enterprise. In the future, case analysis or questionnaire surveys can be combined to deeply understand the specific basic situation and business characteristics of an enterprise and reflect the characteristics and essence of the enterprise.

## Supporting information

S1 Data (XLSX)
